# A human rights approach to an international code of conduct for genomic and clinical data sharing

**DOI:** 10.1007/s00439-014-1432-6

**Published:** 2014-02-27

**Authors:** Bartha M. Knoppers, Jennifer R. Harris, Isabelle Budin-Ljøsne, Edward S. Dove

**Affiliations:** 1 Centre of Genomics and Policy, McGill University, 740 Dr. Penfield Avenue, Suite 5200, Montreal, H3A 0G1 Canada; 2 Division of Epidemiology, Department of Genes and Environment, Norwegian Institute of Public Health, PO Box 4404, Nydalen, 0403 Oslo Norway

## Abstract

Fostering data sharing is a scientific and ethical imperative. Health gains can be achieved more comprehensively and quickly by combining large, information-rich datasets from across conventionally siloed disciplines and geographic areas. While collaboration for data sharing is increasingly embraced by policymakers and the international biomedical community, we lack a common ethical and legal framework to connect regulators, funders, consortia, and research projects so as to facilitate genomic and clinical data linkage, global science collaboration, and responsible research conduct. Governance tools can be used to responsibly steer the sharing of data for proper stewardship of research discovery, genomics research resources, and their clinical applications. In this article, we propose that an international code of conduct be designed to enable global genomic and clinical data sharing for biomedical research. To give this proposed code universal application and accountability, however, we propose to position it within a human rights framework. This proposition is not without precedent: international treaties have long recognized that everyone has a right to the benefits of scientific progress and its applications, and a right to the protection of the moral and material interests resulting from scientific productions. It is time to apply these twin rights to internationally collaborative genomic and clinical data sharing.

## Introduction

In 1948, the General Assembly of the United Nations adopted the *Universal Declaration of Human Rights* (UDHR) to guarantee the rights of every individual in the world. Included were twin rights “to share in scientific advancement and its benefits” and “to the protection of the moral and material interests resulting from any scientific…production of which [a person] is the author” (Art. 27, United Nations 1948). In the 21st century, where are we in realizing the sharing of scientific advancement and its benefits, and the importance of protecting a scientific producer’s moral and material interests? In this article, we argue that these little-developed twin rights, what we call the right “to benefit from” and “to be recognized for”, have direct application to internationally collaborative genomic and clinical data sharing, and can be activated through an international code of conduct.

Sharing genomic and clinical data is critical to achieve precision medicine (National Research Council 2011), that is, more accurate disease classification based on molecular profiles to enable tailored effective treatments, interventions, and models for prevention. Better communication flow across borders and research teams, encompassing data from clinical and population research, enables researchers to connect the diverse types of datasets and expertise needed to elucidate the genomic basis and complexities of disease etiology. Such data integration can make it possible to reveal the genetic basis of cancer, inherited diseases, infectious diseases, and drug responses. Indeed, “[b]y aggregating and analyzing large amounts of genomic and clinical data, it should be possible to discover patterns that would otherwise remain obscure—for example, which mutations within a tumor predict treatment response, or which genetic variants explain rare childhood diseases” (Global Alliance 2013a). Furthermore, integrating genomic data with medical or health records systems securely stored in research and clinical databases, as the eMERGE Network (http://emerge.mc.vanderbilt.edu/) is beginning to do, can help clinicians develop earlier and more targeted treatment strategies for their patients.

The culture of sharing and the development of policies to enable research collaboration are clearly pervasive in genomics research (Budin-Ljøsne et al. 2014; Dyke and Hubbard 2011; Knoppers et al. 2011a) and were propelled by the groundbreaking human genome project (HGP) data release policy, known as the “Bermuda Principles” [Human Genome Organisation (HUGO) 1996]. Data sharing is now emerging in clinical research as well (Mello et al. 2013; Vickers 2006). It is not only driven by numerous international research initiatives (examples can be found in Table 1 of Harris et al. 2012), but also encouraged by many patients and patient organizations, physicians, pharmacists, regulators, and pharmaceutical companies which view data sharing as critical to accelerating research and improving health (http://www.alltrials.net/; http://www.free-the-data.org/; http://www.patientslikeme.com/; http://www.personalgenomes.org/; https://www.reg4all.org/; Kaye et al. 2012; Terry and Terry 2011). In addition, infrastructural initiatives such as P3G’s International Policy interoperability and data Access Clearinghouse (IPAC) (http://www.p3g.org/ipac) work at building internationally interoperable tools for data access and sharing. However, while data sharing and collaboration are increasingly embraced by policymakers, patient advocacy groups and the international biomedical research community, inefficiencies and insufficiencies remain.

## Obstacles to successful global genomic and clinical data sharing

Current data sharing models and policies are not working. The time lags between discovery and translation remain too long, in part the result of ongoing inadequate multidisciplinary collaboration (Contopoulos-Ioannidis et al. 2008). Data are often analyzed in isolated disciplinary or institutional silos and with incompatible methods. Regulatory systems were not designed (nor updated) to foster widespread cross-study collaboration and transborder open sharing of data. “Even where local consent or ethics approval allows data sharing, providing data to researchers in other institutions and countries often requires additional approvals (even when the foreign researchers intend to use the data in a protocol approved by their own local ethics committee)” (Global Alliance 2013a). Relatedly and significantly, there is an absence of universal and enforceable guiding norms that can facilitate an internationally collaborative “harmonizing global science” (Leshner and Turekian 2009) and responsible research conduct. In addition to the problems above, four key obstacles are worth highlighting.

### Lack of policy harmonization

Data sharing is hindered by the lack of harmonization between the policies and procedures of research initiatives (Austin et al. 2012) and of funders. For example, some consortia require a data sharing plan (H3Africa 2013) and data deposit in a public research database [H3Africa 2013; International Cancer Genome Consortium (ICGC) 2012; International Rare Diseases Research Consortium (IRDiRC) 2013], but others do not make such requirements. A further complicating factor is that these data sharing procedures often only apply for the duration of the funded research project, leaving the fate of the data unclear after the collaboration ends (Budin-Ljøsne et al. 2014). Lack of harmonization between biobank practices can also create barriers to data sharing. Examples include special agreements which create limits to data sharing when the policies restrict data release to preferred local research groups (Fortin et al. 2011).

### Lack of structural support

The financial, organizational, technical, and governance structures to support data sharing vary considerably between data sources. While some large community resources benefit from sufficient financial support and solid infrastructures to share data, such as the International HapMap Project (http://hapmap.ncbi.nlm.nih.gov/abouthapmap.html) and the 1000 Genomes Project (http://www.1000genomes.org/), other research projects, while required to share data, have less resources available to do so on a sustainable basis and often lack the administrative and legal support to make the data available (Budin-Ljøsne et al. 2014).

### Legal and ethical hurdles

Even when sufficient resources are in place to share data, legal and ethical hurdles may render data sharing difficult or even impossible (Kuner 2013; Zink and Silman 2008). For instance, the informed consent of the original study may restrict data sharing, or the research ethics committee of the original study may be reluctant to approve data sharing because of privacy and confidentiality concerns (McGuire et al. 2011; Peppercorn et al. 2012).

### Cultural and behavioral considerations

In certain instances, global data sharing could be ethically and legally possible, yet may still be hindered by cultural and behavioral considerations. For instance, research groups in low-income countries may worry that the results derived from data sharing, and the ability to use data and gain normal scientific rewards and recognition, may not be equitably shared with the communities that have contributed data (H3Africa 2013). As a result, researchers may be reluctant to share data they have spent years of effort to collect (Pearce and Smith 2011). The general lack of incentives to share data in a highly competitive environment and the lack of tradition for sharing data outside of one’s own professional discipline can further impede data sharing (Budin-Ljøsne et al. 2014).

To overcome these obstacles, a number of innovative solutions and tools have been proposed. They aim to facilitate the recognition of research contributions, help motivate researchers and the administrators of biobanks to develop and endorse solid data sharing plans, and train junior researchers on responsible data sharing and reuse practices (Harris et al. 2012; Pan et al. 2012). However, these solutions are primarily developed and used only within a few well-connected groups and are not universally applied. These solutions also tend not to be robust enough to adequately address issues related to data sharing that matter to participant-patients and researchers, namely privacy, anti-discrimination and fair access, and proportionate regulatory treatment of research projects. Furthermore, practical tools alone are insufficient to realize the ideal of widespread, international, and cross-disciplinary data sharing.

So how can we work toward a translational, transnational genome science that is cognizant of the need to incentivize data producers to share, to afford researchers proportionate regulatory treatment of research projects, and to address possible patient-participant concerns about the impact of data sharing on privacy, anti-discrimination, and fair access? A new framework is needed that symbolizes both the emerging relationship among 21st century data-intensive science, publics, governments, and research funders, and also the embedding of science in social and political life (Dandara et al. 2012). A human rights framework can offer this.

## What can human rights offer translational and transnational genome science?

Human rights are engrained in international law. They offer many advantages to translational and transnational genome science centered on data sharing, and can better address the data sharing obstacles identified in the previous section. Indeed, their universalizing force can overcome site-specific factors that drive a wedge between research initiative/funder data sharing policies and harmonization. First, because human rights have both political and legal dimensions, they reach beyond the moral appeals of bioethics and can provide a more robust governance framework for the regulation of genomics research (Knoppers 2004). Because they carry international legal force, they can better promote and delineate the contours of responsible access, sharing, and attribution of both research and clinical data. Indeed, if health care becomes a primary location for collecting the phenotypic and genetic data needed to create learning systems for research and clinical care, we need to reinforce the self-regulatory codes of ethics of genomic researchers and clinicians with legally recognized human rights, that is, a *co*-regulatory system.

Second, human rights belong to groups as well as individuals (spurring a reciprocity between the individual and public level) and reach beyond classic negative duties (i.e. forbidding State actors from interfering with the rights of individuals, such as the freedom of expression or the right to privacy) to positive, more progressive duties, urging action by governments (and ideally, industry, funders, and researchers) to share the data, technologies and knowledge that are the fruits of our science to achieve a goal desired by all, such as health (Meslin and Garba 2011).

Another advantage is that human rights can foster responsible translational genomic research by offering stronger protection in three critical areas: privacy; anti-discrimination and fair access; and procedural fairness.

### Privacy

There is concern that sharing certain types of data, particularly identifiable or coded (reversibly de-identified) phenotypic data (which often happens to be clinical data), raises serious privacy problems. Data sharing is not in opposition to privacy and should be conducted in a responsible way such that it does not infringe on the privacy rights of individuals and groups. We believe privacy concerns can be attenuated by human rights protections and robust safeguards built within a principled proportionate governance model (Sethi and Laurie 2013) and a code of conduct that recommends sharing sensitive data within strict limits that respect both laws and ethical guidelines (i.e. sharing data only with “prescribed entities” or within data “safe havens”). As we have argued elsewhere, “despite the perceived ease of re-identification, … technologies and models currently exist that facilitate dissemination of useful health data without compromising privacy” (Knoppers et al. 2012). Self-regulatory instruments and proportionate governance mechanisms like codes of conduct can be human rights attuned and offer privacy protection. In fact, they are supported by the proposed European general data protection regulation, which encourages associations (including funders and research consortia) to draw up codes of conduct that protect the personal data of individuals and confer enforceable rights on them (Art. 38, European Commission 2012; European Parliament 2013).

Further, human rights better protect privacy than status quo informed consent arrangements or bioethical norms alone, for they can (1) hold data users legally accountable, (2) promote feasible, ethically robust standards of data governance, and (3) treat privacy and scientific advancement and its benefits as human rights (not mere interests) to promote, respect, fulfill, *and* balance. While maintaining individual choice models such as informed consent, individual and group privacy concerns, at least those shared by most if not all people, should be assuaged through strong privacy and data security protections beyond informed consent.

Without question, however, the sensitive nature of clinical data remains a challenge, and if we want to achieve a globally collaborative science that harnesses genomic and clinical data sharing, we must strike a careful balance between protecting personal health data from misuse and sharing these data to accelerate health improvements. Nowhere is this more critical than in the genomics context: “At present, it is generally not possible to predict which changes in DNA sequence lead to clinical consequences. Only by comparing each personal genome sequence to a large repository of other such data can robust patterns and relationships be identified. The stakes are high, because if we get it right we can create new opportunities to define diagnostic categories, streamline clinical trials, and match patients to therapy. We want to make sure this is done in a global manner, and with the highest standards for ethics and privacy” (Global Alliance 2013b).

### Anti-discrimination and fair access

Concerns of genetic discrimination or discrimination based on health information are widespread (Otlowski et al. 2012), as are concerns about fair or equitable access to data, research results, and public health benefits derived from those results (de Vries et al. 2011; H3Africa 2013). Anti-discrimination and equitable treatment are also central principles of international human rights law. As Article 2 of the UN’s 1966 International Covenant on Economic, Social and Cultural Rights (ICESCR) obliges States to guarantee that the rights enunciated in the Covenant are exercised without discrimination of any kind, including status, human rights instruments must allow for meaningful sanctions for those who misuse personal data (Joly et al. 2011), including genome sequences that have been volunteered for the public good, for unethical or unlawful purposes, be it discrimination, group stigmatization, misrepresentation, or other negative uses (Editorial 2013). Human rights promote capacity building, which include the sharing of data, knowledge, and resources in currently underserved regions. This can help research projects overcome the persistent obstacle of weak structural support for data sharing, as well as cultural or community concerns that data, scientific rewards, and recognition may not be equitably shared with the communities that have contributed data. As with privacy, treating fair access, anti-discrimination, and harm protection as fundamental principles allows data sharing to be seen as a scientific and ethical imperative (Ohno-Machado 2012) that must always be balanced against competing principles or rights, and not as an unconditional legal duty imposed on any person or entity.

### Procedural fairness

Legal and ethical hurdles in data sharing are often the results of disproportionate research oversight. As a matter of natural justice, and as protected in human rights instruments, there must be fairness in procedures related to regulatory treatment of research projects. In particular, there must be a proportional approach in law or policy guidelines to clearly distinguish between degrees of risk (Dove et al. 2013). Genomic studies or uses of clinical data or samples that involve only non-physical, informational risks should be treated differently (that is, less onerously) than clinical trials with direct physical interventions on the human body. This new informational “*de minimis*” risk category (Rhodes et al. 2011) is suggested for “research that uses genetic information or existing (i.e. stored) biospecimens and [for] research that uses information from databases, medical registries, patient records, and questionnaires or interviews with competent, adult research participants” (Hansson et al. 2013). Such an informational category of “*de minimis* risk” genomic research and medicine would require that personal information be safeguarded from use by those not directly associated with biomedical research and clinical medicine, particularly criminal investigators, insurers, and employers. Furthermore, human rights would necessitate that prospective contributors, users, and authors who are directly associated with the domain abide by ethical access conditions.

## A code of conduct to bridge solidarity human rights to our “social genome”

While the types of human rights invoked in research governance policies vary, often policies have focused on protecting against individual exploitation and harm, rather than on promoting social and economic development that situates genomics research and applications as a public good that enhances human capabilities and economic productivity (Ashcroft 2007). Yet, there is an underlying complementarity between genome science and human rights, especially with social goods-focused human rights and “solidarity rights” that include, for instance, the recognition of the human genome (at the level of the species) as the common heritage of mankind (Knoppers 1991; Kuppuswamy 2009).

Solidarity rights are longstanding in international law. In addition to the 1948 UDHR, the 1966 ICESCR enshrined at art. 15(1)(b) the right “of everyone … to enjoy the benefits of scientific progress and its applications” and the right “of everyone …to benefit from the protection of the moral and material interests resulting from any scientific … production of which he is the author” [art. 15(1)(c)]. Importantly, the ICESCR is legally binding on nations that are States Parties (unlike the 1948 Universal Declaration). They have a duty to respect, protect and fulfill the rights articulated in the ICESCR (Donders 2011). This means that all 160 member nations must promote “the development and the diffusion of science” [art. 15(2)] and “recognize the benefits to be derived from the encouragement and development of international contacts and cooperation in the scientific…field” [art. 15(4)] (United Nations 1966). Moreover, in 2013, an optional protocol entered into force that establishes a complaint mechanism at the international level for people whose rights in the ICESCR have been violated and who have not obtained justice in their own country (United Nations 2008).

These twin rights “to benefit from” and “to be recognized for” are neither self-evident nor promoted in the world of genomics. Indeed, they remain extremely underdeveloped as a whole (Chapman 2009; Donders 2011) and have been cited in only a handful of court decisions around the world, and each time only in passing reference. Yet, from our perspective, the right “to benefit from”, i.e. to enjoy the benefits of scientific progress and its applications, clearly implies a right that all can exercise to have access to and share in both the development and fruits of science across the translation continuum, from basic research through practical, material application (e.g. diagnostics and therapeutics). The right is directly linked to other human rights, especially the right to health, but also to information, expression, association, privacy, and anti-discrimination. As a recent UN report notes, “The ‘benefits’ of science encompass not only scientific results and outcomes but also the scientific process, its methodologies and tools” (Shaheed 2012). And as an international consensus statement from 2009 notes, “this right can be enjoyed individually and collectively” and “implies…scientific freedom, including freedoms of opinion and expression, to seek, receive and impart information, association and movement” (UNESCO 2009). In genomics research, it is a right that all who stand to benefit from genomics can exercise. This right includes opportunities for citizens to take the initiative to share their personal data with the research community (http://www.free-the-data.org/).

Similarly, the right “to be recognized for”, i.e. to benefit from the protection of the moral and material interests resulting from scientific productions, means that all individuals who author or create scientific productions should be acknowledged, and should have their productions valorized if they so choose. It also means they should have their production’s integrity respected. In genomics research, it is a right that all data producers or contributors (e.g. stewards of data repositories) can exercise in the course of data sharing. The right suggests the need for recognition of attribution for data producers and contributors, and perhaps also intellectual property rights. However, as recognized by the UN committee on economic, social and cultural rights, intellectual property rights (such as copyright and patent) must be balanced with the right to enjoy the benefits of scientific progress and its application: “Ultimately, intellectual property is a social product and has a social function. The end which intellectual property protection should serve is the objective of human well-being, to which international human rights instruments give legal expression” (United Nations, Committee on Economic, Social and Cultural Rights 2001).

To apply these twin human rights to translational and transnational genome science, we must move beyond singular policies within funders, research consortia, and biomedical clinical ethics to the meta-level of global governance that works from a universally shared legal, *human rights* framework for addressing international problems. Bioethics norms by themselves have created the necessary foundations to move to this next step (Art. 3 of the Universal Declaration on Bioethics and Human Rights notes that, “Human rights…are to be fully respected”) (UNESCO 2005). However ethical norms alone lack the articulation of the force of governmental and other regulatory stakeholder *duties and standards of accountability*. A human rights perspective can “lay bare the societal importance of the issue and can help mobilize civil society and advocacy groups. It calls for prioritization of the issue on the public policy agenda” (Lemmens 2013). Thus, we suggest looking at tools available in the genomics research governance system, in addition to bioethics norms, that can be used to responsibly steer the sharing and stewardship of research discovery, genomics research resources, and their integration with clinical data for genomic medicine.

It is our contention that regulatory and governance design to enable global collaboration and realization of the twin rights “to benefit from” and “to be recognized for” requires an international code of conduct for genomic and clinical data sharing situated within a human rights framework. It would (a) interpret the right to enjoy the benefits of scientific progress and its applications as being the right to access and share genomic and clinical data across the translation continuum, from basic research through practical, material application (e.g. diagnostics and therapeutics); and (b) apply the right to benefit from the protection of the moral and material interests resulting from scientific productions to genomic research by developing rights such as a right of attribution and a right to integrity of the production for data producers and contributors. Further, this code would establish a set of principles and procedures for responsible research conduct, founded on and guided by complementary human rights principles such as privacy, anti-discrimination, and procedural fairness.

A rights-informed code would be targeted at the structural level of governance frameworks that impose obstacles to data sharing, not at the level of individuals, whom we stress are not obligated to share genomic or clinical data. Choice remains primary: “The right to share in scientific benefits should not be predicated on participation” (UNESCO 2009). State governments are the principal, but certainly not only, entities responsible for the promotion and protection of international human rights, and therefore should sign onto a code to commit themselves to three duties. They must *respect* these twin rights, i.e. “respect the freedom of the scientific community and its individual members to collaborate with others both within and across the country’s borders, including the free exchange of information, research ideas and results” (UNESCO 2009). They must *protect* these twin rights, i.e. pass laws or regulations that ensure that private non-State parties do not violate the rights, including a right of attribution of data contributors or producers. And they must *fulfill* these twin rights, i.e. undertake an obligation to progressively make the rights a reality through infrastructural and institutional measures that promote data sharing.

The human rights framework, with its universal scope and legally enforceable mechanisms adhered to by State and non-State actors, can effectively regulate behavior and set norms as genomic data are joined with clinical data to create international databases for researchers and clinicians to access such data. In addition to the protections discussed above, this approach would also protect patient-centric systems of dynamic participation that provide information and support resources for the individual contribution of sensitive data (medical, genetic, etc.) for research use around the world (Terry et al. 2013). The human rights framework would help to achieve the realization of the “social genome” (Knoppers and Joly 2007) under international law, and can provide the appropriate mechanisms for ensuring respect of the ethical principles (Chapman and Wyndham 2013) that have framed the self-regulatory norms of genomics research to date. This contention is supported by a UN report, which notes that: “[d]eveloping codes of conduct explicitly informed by human rights…seems essential” (Shaheed 2012).

### Possible objections to a human rights framework

Situating a code of conduct within a human rights framework faces some objections. First, some may argue that human rights or international codes or declarations can only be, at best, an amalgamation of minimalist or vague claims to achieve international consensus, or, at worst, merely a culturally imperialist Western ideological construct (Benatar 2005). We think, on the contrary, that all people are entitled to basic rights by the mere fact of their human condition. To be human is to possess certain *fundamental* rights, regardless of one’s status. Let us recall that essentially all of the world’s countries have ratified the UDHR, which itself was drafted by members composed of varying religious and philosophical and cultural traditions, and 160 countries have ratified the ICESCR. The fundamental rights contained in the UDHR and ICESCR are not imposing one *cultural* standard. Rather, they are setting a baseline *legal* standard that all individuals and peoples can invoke to ensure basic human dignity and, quoting the Preamble of the UDHR, to establish the conditions “for freedom, justice and peace in the world” (United Nations 1948). Also, rather than being culturally imperialist, human rights entail international cooperation: “It is particularly incumbent upon those States which are in a position to assist others” to help provide access to and use of science and technology (United Nations, Committee on Economic, Social and Cultural Rights 1990). Responsibly sharing genomic and clinical data around the globe is an important component of international cooperation and assistance that reflects not a Western ideological construct but a belief in human solidarity.

Second, some may argue that human rights, and more broadly, international law, face accountability, monitoring, and enforcement problems. Short of binding law, how can codes of conduct be governed and enforced effectively? While it may be true that individuals or communities cannot always directly enforce or invoke in a court of law the twin rights “to benefit from” and “to be recognized for,” these rights exist nonetheless; States are legally bound to implement them and non-State parties are bound to respect them. One solution alluded to above could be that governments or regulatory agencies endorse a code of conduct, similar to what they do for binding corporate rules (BCRs) (Moerel 2012), and what the European Commission aims to do in its proposed General Data Protection Regulation (Art. 38, European Commission 2012; European Parliament 2013). This would put the obligation to monitor and enforce codes on governments, supervisory bodies, and non-governmental organizations alike. Moreover, funding agencies and even industry could make compliance with codes of conduct a special term and condition in notices of award or in contracts. Failure to comply with a code could lead to enforcement actions such as funding withholds or termination, as well as remedies for those individuals or groups whose access rights have been infringed or who face breaches to privacy, discrimination, or violations of procedural fairness.

## Conclusion: toward a code of conduct for global genomic and clinical data sharing

This article has advocated for a durable, communal, and purpose-driven code of conduct set within a human rights framework that both realizes and maximizes the sharing of scientific progress and its applications for all while providing proper attribution for scientific producers. We believe that in so doing, a code can set an innovative but effective and ethically responsible approach to sharing genomic and clinical data. It can help move us beyond risk-focused principles and policies to a framework that views genomic and clinical databases as global public goods (Human Genome Organisation (HUGO) 2002) that must be respected, protected, and promoted, and that draws a roadmap for conducting collaborative genome science in an ethically responsible, solidaristic manner. It will also elaborate and clarify the normative content of rights and the corresponding obligations of parties. This allows “individuals and communities [to] learn what they are legally entitled to, States [to] know what kind of legal obligations they have in relation to the implementation of these rights and supervisory bodies [to] monitor the performance of States in this regard” (Donders 2011).

Knowledge production is increasingly conducted in teams (Wuchty et al. 2007). A global alliance of international partners, from research organizations to funders to industry and governments, must work together to create conditions for the availability, accessibility, and acceptability of quality genomic and clinical data that improve human health. Global governance “must take root ab initio and be co-produced as part of a larger, collaborative, co-evolving social structure that includes data-intensive science, technology, and genomics medicine” (Dandara et al. 2012). Only by working together to develop common principles, as well as more practical policies and procedures, can patients, participants, researchers, and clinicians safely and effectively share data, protect and promote privacy, and foster medical progress across the globe.

Therefore, building on a genomic data sharing code of conduct we previously proposed (Knoppers et al. 2011b), we will now endeavor to design a Code that is actionable and applicable and practicable for funders, regulators, researchers, clinicians, and patient-participants using genomic and clinical data. We will engage stakeholders around the world over the coming year to help co-design this Code. In the spirit of collaboration, and to honor the principle of citizenry, we encourage the journal’s readership, as well as the broader public, to send us questions and comments and work with us on this important policymaking project.
